# Highlighted Advances in Therapies for Difficult-To-Treat Brain Tumours Such as Glioblastoma

**DOI:** 10.3390/pharmaceutics15030928

**Published:** 2023-03-13

**Authors:** Nuno Cruz, Manuel Herculano-Carvalho, Diogo Roque, Cláudia C. Faria, Rita Cascão, Hugo Alexandre Ferreira, Catarina Pinto Reis, Nuno Matela

**Affiliations:** 1 Instituto de Biofísica e Engenharia Biomédica, Faculdade de Ciências, Universidade de Lisboa, 1749-016 Lisboa, Portugal; 2 iMED.ULisboa, Research Institute for Medicines, Faculdade de Farmácia, Universidade de Lisboa, Av. Prof. Gama Pinto, 1649-003 Lisboa, Portugal; 3 Instituto de Medicina Molecular João Lobo Antunes, Faculdade de Medicina, Universidade de Lisboa, 1649-028 Lisboa, Portugal; 4 Department of Neurosurgery, Hospital de Santa Maria, Centro Hospitalar Universitário Lisboa Norte (CHULN), 1649-028 Lisboa, Portugal

**Keywords:** brain tumour, glioblastoma multiforme, treatment, imaging, nanotechnology, tumour treating field

## Abstract

Glioblastoma multiforme (GBM) remains a challenging disease, as it is the most common and deadly brain tumour in adults and has no curative solution and an overall short survival time. This incurability and short survival time means that, despite its rarity (average incidence of 3.2 per 100,000 persons), there has been an increased effort to try to treat this disease. Standard of care in newly diagnosed glioblastoma is maximal tumour resection followed by initial concomitant radiotherapy and temozolomide (TMZ) and then further chemotherapy with TMZ. Imaging techniques are key not only to diagnose the extent of the affected tissue but also for surgery planning and even for intraoperative use. Eligible patients may combine TMZ with tumour treating fields (TTF) therapy, which delivers low-intensity and intermediate-frequency electric fields to arrest tumour growth. Nonetheless, the blood–brain barrier (BBB) and systemic side effects are obstacles to successful chemotherapy in GBM; thus, more targeted, custom therapies such as immunotherapy and nanotechnological drug delivery systems have been undergoing research with varying degrees of success. This review proposes an overview of the pathophysiology, possible treatments, and the most (not all) representative examples of the latest advancements.

## 1. Introduction

Glioblastoma multiforme (GBM) is the most common brain tumour (in adults), and it is characterized by being very aggressive, incurable, and having a low median survival of a year to two years depending on the time of diagnosis, prognostics, and applied treatment [1]. According to the 2016 World Health Organization (WHO) classification of central nervous system tumours (CNS), GBM is classified as a grade IV glioma [2]. In the newest 2021 WHO classification, the designation glioblastoma now refers only to CNS grade IV diffuse and astrocytic isocitrate dehydrogenase (*IDH*)-*wildtype* glioma in adults. Grade IV *IDH-mutant* manifestations in adults are referred as grade IV astrocytoma, *IDH-mutant*, whereas paediatric diffuse gliomas are classified in low- or high-grade families, subdivided according to histological and molecular characteristics [3]. To retain consistency with the newest classification while being able to analyse material using the older nomenclature, the deprecated term “GBM” will be used, but it will not be used interchangeably with “glioblastoma”, which will be used according to the 2021 WHO classification. 

The disease is poorly understood, and it seems there is not a clear cause since neither family history nor critical germline alterations are prevalent in these patients [4]. Symptoms of GBM vary, depending on the size of the tumour and brain area affected, ranging from persistent, overall weakness, to nausea, seizures, and memory disorders [4,5]. Symptoms and/or the presence of a suspected lesion in a medical image of the brain precede diagnosis via histological and genetic studies of the tissue obtained through biopsy and/or resection [6]. These assays are paramount since the characteristics of the tumour influence the therapy that is prescribed; tumours with *IDH 1* or *IDH 2* mutations are less aggressive and respond better to radiotherapy followed by chemotherapy, whereas methylation of the O6-methylguanine deoxyribonucleic acid (DNA) methyltransferase (*MGMT*) gene predicts a positive response to chemotherapy with alkylating agents [7].

As the most common type of primary malignant brain tumour, with an average incidence of 3.2 per 100,000 persons, GBM makes up 54% of all gliomas and 16% of all brain tumours, and it presents at a median age of 64 years [8,9]. Incidence also increases with age up to 15 per 100,000 persons for the 75–84 age group. Males are 1.6 times more likely to develop the disease than females [8,10].

The poor prognosis of this tumour is tied to its capability to persist even after tumour resection and therapy; tumour removal is very difficult due to the tumour’s infiltrative nature and how sensitive healthy brain tissue is to surgical intervention, risking loss of any function. Furthermore, GBM responds poorly to chemotherapy and radiotherapy, as it is able to attain resistance to chemotherapy, whereas radiotherapy can cause necrosis of healthy tissue, risking further loss of function as well [11,12].

There have been attempts to overcome these difficulties, both in improving diagnosis and characterization of the tumour area for higher resection and relying on innovative therapeutic strategies. Immunotherapy and nanotechnology are such examples. In this review, some of these recent advancements, as well as the standard of care in GBM therapy, are described. With such an overview, we aim to provide broad information through which the highlighted advances can be contextualized. This overview includes works published in the last 10 years (available via PubMed and indexed in MEDLINE) with either glioblastoma or GBM in their keywords and/or title. In addition, works featuring adult glioblastoma and clinical trials with an associated publication were also analysed and included. In particular, a set of clinical trials registered at ClinicalTrials.gov, which is the world’s largest clinical trial registry, was analysed.

## 2. Treatment

**Figure 1:**
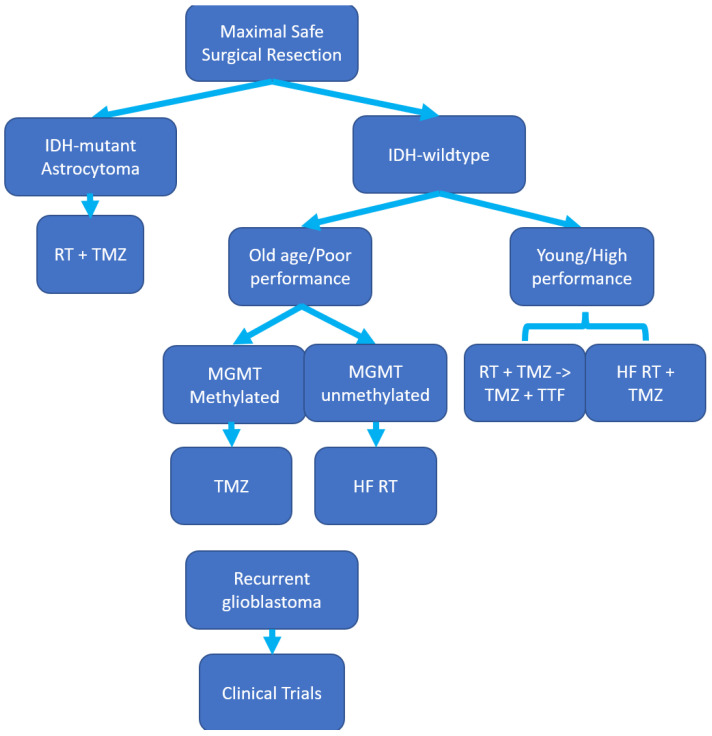
Therapeutic approaches to high-grade glioblastoma multiforme (GBM). IDH, isocitrate dehydrogenase; performance, Karnofsky Performance Score (KPS); MGMT, O6-methylguanyl DNA methyltransferase gene promoter; TMZ, temozolomide 150–200 mg/m^2^ on 5/28 days; RT + TMZ, radiotherapy 30 × 2 Gy with daily concomitant temozolomide at 75 mg/m^2^; HF RT, hypofractionated radiotherapy, 15 × 2.66 Gy; TTF, tumour treating fields.

Currently, the standard of care for GBM therapy consists of maximal safe surgical resection, followed by radiotherapy (RT) and chemotherapy, with the recent addition of tumour treating fields (TTF) therapy [7,11,13]. A schematic representation of current treatment recommendations according to the American Society of Clinical Oncology and the Society for Neuro-Oncology (ASCO-SNO) guidelines is presented in Figure 1 [14].

The 2021 European Association of Neuro-Oncology (EANO) guidelines are similar to the ASCO-NSO guidelines, but they differ by not including TTF and recommending various options besides experimental treatment in recurring GBM (repeat surgery, alkylating chemotherapy, bevacizumab, and re-irradiation) [15]. However, these are to be chosen depending on patient characteristics (Karnofsky Performance Score, *MGMT* methylation, prior therapy, and age) and do not state them as standard of care [15].

### 2.1. Surgery

Surgery aims to not only provide relief to the patient from the effect of the growing cancerous mass but also to obtain material for pathological analysis [16]. Preoperative evaluation of the patient involves anatomic and metabolic characterization of the lesion via medical imaging techniques [9,17,18]. An overview of some of the techniques used in this evaluation will now follow.

#### 2.1.1. Preoperative Techniques

The preoperative techniques here presented rely on imaging techniques to obtain anatomical and physiological data on the tumour, “mapping” the target volume by revealing tumour mass spread, and detailing its location in relation to important, eloquent brain tissue.

Magnetic resonance imaging (MRI), which uses strong magnetic fields and radio waves to excite protons and produce an image [19], is the standard method of lesion visualisation. Computed tomography (CT), a computerized X-ray imaging technique which uses narrow X-ray beams quickly rotated around the body to produce cross-sectional images, is used when MRI is not available or possible (e.g., a patient with a non-MRI-compatible pacemaker or other metallic implants) [17]. Gadolinium enhancement is a typical feature of GBM, and this imaging technique usually reveals a heterogeneous mass with a necrotic core and surrounding oedema [9,20]. Magnetic resonance spectroscopy (MRS) allows for further characterization of the lesion by visualising metabolites that can be associated with a malignant tumour, specifically an increase in choline and decrease in N-acetyl aspartate, which are associated with higher cellular turnover and lower neuronal cellularity [18,20]. It also allows primary brain tumours, radiation necrosis, and metastases to be distinguished [21].

Positron emission tomography (PET) is another imaging technique that retrieves metabolic information, as it can measure the uptake of a radioactive tracer into the tissues of interest [22]. After administration of the tracer, a biologically active molecule such as an amino acid or carbohydrate [23], and waiting for its uptake, the patient is taken to a scanner [22]. Then, as the name implies, the tracer decays, emitting positrons that annihilate themselves with electrons of the tissues, producing 511 keV photons [24]. These photons can then be detected using appropriate equipment, and an image can be produced [24,25]. In particular, ^18^Fluoro-O-(2) fluoroethyl-l-tyrosine([^18^F] FET) or FET is commonly used due to its longer half-life compared to alternatives such as Carbon-11 and due to its sensitivity and specificity to GBM; it is thus able to improve glioma identification, tumour diagnosis, and size assessment [26,27]. When combined with MRI, FET PET can allow for better evaluation of progression and response, as contrast is not affected by changes in the blood–brain barrier (BBB) that can be caused by therapy [26,28,29]. These traits mean that FET PET has prognostic value in radiotherapy planning and results in better tumour tissue delineation for surgical planning [30,31,32]. FET PET’s properties have led to interest in hybrid imaging systems, such as MR-PET. The combination of these imaging techniques improves tumour delineation compared to MRI, supplying metabolic and anatomical data invaluable to biopsy and surgical planning [33,34,35]. Minimizing intermodal interference is a main concern when designing an MR-PET system, and efforts in PET component shielding, MR magnetic field calibration, and optimization of data acquisition and image reconstruction have been made to unlock this technique’s potential [36].

Tractography is a way to analyse the brain’s white matter in 3D space [37]. It is an MRI sequence that calculates water diffusion and detects the Brownian motion of water inside cells, creating a map of white matter tracts [37,38]. This technique allows for better planning of surgery to avoid possible eloquent white matter tracts, which maximises resection and preservation of function [39]. It can be combined with preoperative blood-oxygenation-level-dependent (BOLD) functional MRI (fMRI) to complement with information on areas dedicated to speech and movement, as well as hemispheric dominance [37,38]. This combination can further increase the amount of eloquent tissue spared [39,40]. The anatomical and functional data provided by these techniques is then used to plan a surgical intervention.

#### 2.1.2. Intraoperative Techniques

Medical imaging techniques are also applied during surgery: these intraoperative imaging techniques aim to assist the surgeon to achieve maximum resection while preserving eloquent areas of the brain [11,18,41]. One such technique applies a fluorescent agent called 5-aminolevulinic acid (5-ALA) to achieve a red coloration of tumour tissue under blue light stimulation (400–410 nm) [42], which allows for a greater extent of resection when compared to surgery under white light [43]. The biggest limitation of this technique is that direct visualisation of the tissue is required, so additional functional and anatomical information of the tumour is invaluable to avoid both a bad estimation of total gross resection and damage to eloquent areas [18]. An alternative but not routine technique aimed at preserving eloquent areas uses another fluorescent agent, indocyanine green (ICG), to produce videoangiography of cerebral vessels, thus allowing the surgeon to avoid injuring small calibre vessels during resection [41]. Intraoperative MRI (iMRI) is the utilization of MRI during surgery to acquire real-time, high-definition images that allow the surgeon to evaluate the extent of resection more easily via a portable device or moving the patient to a nearby iMRI room [44]. Evidence on the usefulness of iMRI is split. On one hand, there is some evidence that iMRI is helpful, as it circumvents the limitations of preoperative imaging, which does not translate anatomy as well due to brain volume distortions after craniotomy [45]. On the other hand, it is a costly technique that does not show any statistically relevant difference compared to less advanced techniques, namely 5-ALA [38,46,47]. That said, there has been some evidence that the combined use of iMRI and 5-ALA achieves the best possible results in the extent of resection without any significant increase in post-operative neurological deficits [48,49].

#### 2.1.3. Pseudoprogression

Distinguishing true tumour progression (TP) from pseudoprogression (PsP) is a challenge given that medical imaging is both used in the execution and interpretation of clinical trials and in day-to-day patient evaluation [50]. Pseudoprogression usually manifests as posttreatment radiographic evidence of disease progression that can be accompanied by symptoms, which resolves or improves without additional treatment [51,52]. To improve diagnosis, both the establishment of predictive factors and improvement of imaging techniques are necessary. In a literature review by Fèvre et al. [53], the literature featuring PsP in patients treated with chemoradiation was examined to identify clinical and molecular markers to differentiate PsP from TP. They concluded that the data are discordant and that the current standard, response assessment in neuro-oncology (RANO) [54] and modified RANO [55], remains the most applicable in clinical practice. Other reviews [50,51,56,57,58] focus on MR and PET approaches to PsP in brain tumours and agree that advanced imaging techniques such as diffusion-weighted imaging, perfusion MRI, single photon emission computed tomography, and proton MRS are better at distinguishing PsP from TP than conventional MRI. However, these reviews also point out a lack of standardization of methodology for data acquisition as well as a lack of large validation studies.

#### 2.1.4. Recent Advances

In the field of GBM imaging, Denora et al. [59] synthesised ultra-small iron oxide nanoparticles (USPIONs) combined with a fluorescent dye and a translocator protein (TSPO) ligand for near-infrared (NIR) imaging. TSPO is overexpressed in several cancer types, including gliomas. The system showed no toxicity in vitro, and selective targeting of the nanoparticles to TSPO was confirmed both in vitro and in a murine xenograft model [59]. Henderson et al. [60] explored the potential role of desorption electrospray ionisation mass spectrometry (DESI-MS) for ex vivo sample analysis. They constructed 3D DESI-MS images by combining the 2D data, which allowed them to obtain a 3D representation of the sample that evidenced its metabolic heterogeneity. They posit that this method of automated 3D DESI-MS acquisition can increase throughput for larger amounts of samples and provide important information about GBM responses to its microenvironment and hypoxia [60].

Recent advances in PsP identification include work by Sun et al. [61], who used a machine learning strategy combined with radiomic features from T_1_-weighted contrast-enhanced imaging to differentiate PsP from TP. GBM patients that underwent total or subtotal resection and chemoradiotherapy and were confirmed via second surgery or radiological follow-up to exhibit either TP or PsP made up the training population. Volume of interest was determined separately by two experienced (10+ years of experience) neuroradiologists, with a third senior (25 years of experience) neuroradiologist resolving inconsistencies between their determinations. The research group found that, when compared to the performance of three junior (7–8 years of experience) neuroradiologists, the radiomics model showed better classification performance in accuracy, sensitivity, and specificity. Limitations of this study include a small sample size (77 patients) and no consideration for the genetic markers published in the 2016 WHO classification of glioblastoma. 

Other studies investigated additional techniques that go beyond the use of imaging and approach the surgical procedure itself. Gerritsen and colleagues [62] investigated the use of awake craniotomy, where the patient is kept awake so cortical mapping of eloquent structures can be made, versus surgery under general anaesthesia. Researchers found that for low-grade glioma, awake craniotomy resulted in greater resection, lower neurological complications, and better quality of life for patients, with recent results for GBM also appearing promising. In this research field, Dimou et al. [63] conducted a systematic review of the effects of supramaximal resection, where the surgeon removes tissue beyond the T1-enhancement area provided by MRI, to tackle GBM’s ability to spread microscopically beyond the macroscopically observable border. However, no strong evidence towards the usefulness of this technique was found in their analysis. Bettag et al. [64] studied the use of an endoscope to induce fluorescence in 5-ALA fluorescence-guided resection, which allows a surgeon to observe the target tissue without needing direct line of sight. They administered a standard dose of 5-ALA (20 mg/kg) and used a prototype endoscope to scan for any remaining fluorescent tissue after normal fluorescence-guided resection; they achieved an increase in resection rate from 65% to 95%, although the study was limited by the small sample of twenty patients.

### 2.2. Chemotherapy

#### 2.2.1. Standard of Care

After maximal resection of the cancerous mass, the next treatment involves concomitant radio and chemotherapy. The standard of care is a 60 Gray (Gy) dose of radiation, fractionated in portions of 2 Gy, with daily administration of temozolomide (TMZ) at 75 mg/m^2^ during radiotherapy, followed by six maintenance cycles of 150 to 200 mg/m^2^ of TMZ for 5 days every 28 days [7,65,66]. TMZ is an alkylating agent that is administered orally, which works by alkylating/methylating guanine residues in DNA, thus leading to tumoral cytotoxicity through cell cycle arrest [11,67]. A 5-year study of the effects of TMZ on overall survival (OS) of GBM patients found a significant increase compared to radiotherapy alone: from 10.9 to 27.2% 2-year survival [68]. However, TMZ is limited in its action due to GBM’s capability to develop resistance to the drug. Basically, if tumour cells retain their ability to repair the damage done by methylation of DNA, they can overcome TMZ treatment [69]. One such repair mechanism is regulated by *MGMT*, a DNA repair enzyme, and it has been shown that patients with a methylated *MGMT* promoter gene had a better prognostic and OS than patients with the non-methylated *MGMT* promoter gene [68,69,70].

Another drug used in GBM treatment is carmustine. It is a nitrosourea with alkylating and BBB-crossing capabilities [71]. It is usually administered in a polymeric wafer (carmustine wafers, CW) implanted after tumour resection. [20,67,72]. This local administration of carmustine greatly reduces the severe toxic effects of the drug, namely bone marrow suppression, hematotoxicity, hepatotoxicity, nephrotoxicity, and pulmonary fibrosis. Such toxicity is the reason why carmustine use was avoided after the introduction of TMZ to the treatment of GBM by the Food and Drug Administration (FDA) in 1999 [73]. Such delivery of the drug as CW allows for a 100-fold increase in the local concentration without the systemic side effects of intravenous administration [74]. Nonetheless, the use of CW remains controversial due to the drug’s toxicity, the potential for adverse side effects, and post-surgery complications and infections in the case of wafer usage, though recent studies show an increase of median survival to about 17 months [75,76]. 

Bevacizumab, like carmustine and TMZ, is an FDA-approved chemotherapeutic agent for the treatment of GBM, which has been used since 2006. It consists of a monoclonal antibody that targets the vascular endothelial growth factor (VEGF) ligand, thus inhibiting tumour-driven angiogenesis [67,70]. When administered in conjunction with the standard-of-care treatment, bevacizumab increases progression-free survival (PFS) but not OS [11,13,67,77]. The main adverse side effects are arterial hypertension, although there are other rarer but serious effects such as haemorrhages, thromboembolic events, complications of wound healing, congestive heart failure, and gastrointestinal perforations [9,17]. The 2022 ASCO-SNO guideline does not consider bevacizumab’s benefits to outweigh the harms, so this drug is no longer recommended for GBM treatment [14]. Nonetheless, the EANO guidelines recognize bevacizumab as an option for recurrent disease and symptomatic patients with large tumours, although it is not approved for patients in the European Union [15].

#### 2.2.2. Nanomedicine in GBM Chemotherapy

To overcome both the physical barrier to treatment that is the BBB and to reduce side effects from treatment, nanotechnology has been applied in drug delivery. Nanotechnological drug delivery systems have seen use in different applications from dermo-cosmetic, to wound healing, and therapies such as photodynamic therapy (PDT) [78,79,80,81]. Recent efforts in GBM chemotherapy include work from Guo et al., where a thiolated paclitaxel-oligo (p-phenylene vinylene) nanomedicine was designed [82]. The authors suggested that this nanocarrier was able both to cross the BBB (10% BBB penetration) and form aggregates around the tumour, thus reducing payload escape from the target tissue. This nanocarrier led to at least 3% more apoptosis of U87MG and U343 GBM lines when compared to free paclitaxel (PTX). Moreover, selective retention on GBM since the first hour of treatment was observed, and it remained on GBM tissue even after seven days. Moreover, the in vivo inhibition of GBM exceeded 50%, which is greater than the 26% inhibition rate of free PTX, and it did not induce any weight loss. 

Another successful example was published by Ferreira et al. [83]. They designed chitosan-poly(lactic-co-glycolic acid) (chitosan-PLGA) nanoparticles with mucoadhesive properties for nose-to-brain delivery of chemotherapy (alpha-cyano-4-hydroxycinnamic acid (CHC) and the monoclonal antibody cetuximab (CTX)), thus bypassing the BBB. These particles were stable for up to 3 months and would only induce cytotoxicity when carrying the therapeutic agent. Their mucoadhesive particles were stable, with a positive surface charge (+37 mV) and a high entrapment efficiency (>75%). The chitosan-PLGA nanoparticles themselves did not incur any in vitro cytotoxic activity when loaded with CHC and surface functionalized with CTX. They dropped cell viability of human astrocytoma model SW1088 and human glioblastoma model U251 to around 5%.

Zheng et al., designed a self-assembling protein structure of haemoglobin and glucose oxidase encapsulated by a red blood cell membrane [84]. This biocompatible nanosystem was found to be capable of crossing the BBB and accumulating at the tumour site, thus generating anti-tumour activity via reactive oxygen species (ROS). The nanosystem exhibited high in vitro cytotoxicity towards the GBM cell line U87MG and murine fibroblast NIH-3T3 cells but only when glucose was present to allow for ROS production; it dropped cell viability to around 20%. In vivo studies also confirmed BBB crossing and passive targeting of cancer tissue; 12 h after intravenous administration of ICG-loaded nanoparticles, fluorescence in brain tissue was evident. After 72 h, this fluorescent signal remained in the brain, whereas it was absent in other major organs. Furthermore, treatment supressed tumour growth in mice and induced apoptosis of cancer tissue.

Saha et al., designed amphetamine lipid nanoparticles, exploiting the innate capabilities of amphetamines to cross the BBB [85]. These loaded nanoparticles were not only non-cytotoxic but also showed preferential accumulation in the brain and were able to encapsulate PTX and programmed death-ligand 1-small interfering RNA (PDL1-siRNA), two anti-glioma drugs. Controlled release of PTX was also achieved after 25 h, with the release at physiological pH being lower (<60%) than at a pH of 5 (>80%). In vitro cell viability studies also showed that cytotoxicity towards non-tumoral model Chinese hamster ovary cells of PTX-loaded nanoparticles was around 20%, whereas against the GBM cell line GL261, it ranged from 50 to 70% after 24 h. This study showed selective uptake and efficacy. Finally, in vivo tests with C57BL/6 mice bearing GL261 GBM showed that PTX-loaded nanoparticles increased median OS from 15 to 25 days. However, when co-loading an immune checkpoint inhibitor (PDL-1 siRNA) with PTX, median OS increased to 45 days. No in vivo toxicity or alteration of haematological and biochemical markers was observed when the treatment was administered to non-glioblastoma-bearing mice.

Manju and colleagues [86] produced a multi-gene-targeted siRNA nanoparticle gel (NPG) for intracranial injection. The genes targeted were *FAK*, *NOTCH-1,* and *SOX-2* expressed in glioma stem cells (GSCs). The gel was prepared by the self-assembly of siRNA with a protamine–hyaluronic acid combination, resulting in siRNA nanoparticles with an average size of 250 nm as determined by dynamic light scattering (DLS) and 95% loading efficiency as determined by agarose gel electrophoresis. The group justified the triple gene targeting with these genes’ inter-regulation, which makes single-gene-targeting therapy insufficient. In vitro, rat glioma neurosphere formation after 14 days was suppressed by 90% compared to untreated cells and compared to 20% suppression for TMZ-treated cells. Furthermore, for human GBM neurospheres, TMZ did not supress neurosphere formation, whereas NPG-treated cells achieved over 90% suppression. In vivo, in a patient-derived xenograft model, the treated group was administered NPG at the date of tumour transplant (day seven), which showed no significant tumour growth after 120 days, as confirmed through MRI. The control recorded tumour volume was 2.5 cm^3^ by day 30, and TMZ-treated rats exhibited 1.5 cm^3^ tumour volumes. Most importantly, intracranial administration of NPG in a rat orthotopic tumour model (1.9 mm^3^ tumour volume confirmed by MRI) exhibited no tumour growth up to 120 days after a single dose.

### 2.3. Radiotherapy

As mentioned in the previous section, the standard of care after maximal resection is concomitant fractionated RT (60 Gy given in fractions of 2 Gy over 6 weeks) with TMZ [67]. RT achieves its therapeutic action by damaging tumour cells’ DNA and creating free radicals via ionizing radiation [87,88]. However, GBM is capable of recurrence after therapy, with the possibility of developing resistance to RT [88,89]. Nonetheless, different strategies for GBM RT exist, from typical 3D conformal RT, where X-rays are delivered to the target volume from different angles to spare non-malignant tissue, to stereotactic radiosurgery (SRS), where focused beams achieve more precise targeting than 3D conformal RT but are limited to smaller volumes and require patient immobilization, or brachytherapy, where radioactive vectors are implanted into the tumour bed [90,91]. Proton therapy (PT) or proton beam radiation therapy (PBRT) is yet another strategy that utilizes protons instead of photons for focused energy delivery to target volume, exploiting the faster deceleration of protons. This allows for limited off-target doses compared to photon RT [92].

Recent advances include a phase II trial published by Mohan et al. [93], where they investigated the effect of proton vs. photon radiotherapy in radiation-induced grade 3+ lymphopenia (G3 + L). Both arms in this randomized trial were treated with concomitant TMZ and had their absolute lymphocyte count (ALC) measured before, during, and up to 1 month after treatment. Researchers found that males and individuals treated with PT had lower incidence of G3 + L (15 and 14%, respectively) versus X-ray radiotherapy (XRT) (39%) and that individuals treated with PT had a lower whole-brain mean dose (WBMD) (20.1 ± 5.7 Gy) compared to XRT (27.0 ± 6.1 Gy). 

In another phase II trial involving PT [94], Brown and colleagues compared time to cognitive failure of patients treated with intensity-modulated radiotherapy (IMRT), an XRT modality where increased 3D conformity is achieved via several modulated beams with different intensities, at different angles, versus PT. All patients were newly diagnosed with GBM and randomized unblinded 1:1 to each group. The primary endpoint was time to cognitive failure, with secondary endpoints being overall survival (OS), intracranial progression-free survival (PFS), toxicity, and patient-reported outcomes (PROs). The study found there was no statistical difference in OS (median 21.2 months in IMRT vs. 24.5 months in PT), PFS (8.9 months in IMRT vs. 6.6 months in PT), or rates of deterioration between the two arms at 6 months. There was a reduction in mean number of toxicities (1.15 IMRT vs. 0.35 PT) and patient reported fatigue (58% IMRT vs. 24% PT). The study was limited by its sample size and the number of patients from the PT arm that were denied treatment by their insurance, which further reduced the size of the arm.

### 2.4. Immunotherapy

By a very short definition, cancer immunotherapy is the activation of the immune system to fight cancer cells, and it has been used against melanoma, prostate, and lung cancers, among others [95]. There are several approaches to immunotherapy, from vaccination, which exposes the immune system to tumour-specific antigens; to adoptive cell therapy (ACT), where specific antigen receptors are introduced into T cells, creating T cells specifically capable of targeting tumour cells; the inhibition of tumour immunosuppressants (check-point inhibition); and the stimulation of the immune system with cytokines such as interleukine-2 (IL-2) [67]. The biggest hurdle that must be overcome for GBM immunotherapy is its capability to avoid the immune response [13,96,97]. Low T cell infiltration due to the BBB, no lymphatic drainage, low antigen presentation, and high heterogeneity all facilitate its immune evasion [13,98,99]. Furthermore, it has been found that GBM patients tend to exhibit an increased Treg (an immunosuppressant T cell) population, and the glioma itself has the ability to produce and secrete immunosuppressants and immune response downregulators such as interleukin-10 (IL-10) and indoleamine-pyrrole 2,3-dioxygenase (IDO) [97]. These difficulties mean that there is no standard-of-care immunotherapy for GBM yet.

A systematic review by Marenco-Hillembrand et al., in 2020 found that cancer-vaccine-based immunotherapy has shown median OS increases, though only dendritic vaccines were able to provide any significant increase, albeit a marginal increase, when compared to the standard of care [100]. Youssef and colleagues reviewed the role of Ipilimumab in GBM immunotherapy. Ipilimumab is a monoclonal antibody that targets the cytotoxic T-lymphocyte-associated protein 4 (CTLA-4) checkpoint, a downregulator of the immune response that is currently used to treat skin, prostate, and lung cancers. This review concluded that monotherapy with the drug has not yet delivered outside of preclinical trials with animal models; however, co-adjuvant therapies exhibit promising results [101]. In a different point of view, the use of viral vectors to either deliver a genetic payload or selectively lyse tumour cells has also seen important developments with Ofranergene obadenovec (VB-111) in combination with bevacizumab. A phase III trial compared this combination against bevacizumab monotherapy. However, no statistically significant improvement in OS was observed [102]. Another phase III study, with the programmed cell death protein 1 (PD-1) immune checkpoint blockade nivolumab showed no statistical difference in OS when compared with bevacizumab [103]. Haddad et al., attributed the contrast between the successes in preclinical trials and the failures in later phase clinical trials to the difficulty of creating a model that allows for a holistic representation of the immune system’s response to GBM [104]. 

In recent developments, Wang et al. [105] produced a pH-sensitive BBB-crossing protein delivery and release system for anti-PD-L1 (programmed death ligand 1). The nanosystem relies on the choline analogue, 2-methacryloyloxyethyl phosphorylcholine (MPC), polymerized with short-chained poly(ethylene-glycol) methacrylate (PEGMA) to form a precursor, which is then conjugated with anti-PD-L1 via the pH-sensitive linker 3-(bromomethyl)-4-methyl-2,5-furandione. This choline analogue system targets the choline transporter expressed on the endothelial cells that constitute the BBB, and the pH-sensitive component releases the anti-PD-L1 in the acidic tumour environment. The group obtained spherical particles with sizes under 100 nm, which are stable in cell culture medium solutions. In a BBB in vitro model, a maximum 4 h penetration efficiency of 12% was achieved. In vitro cytotoxicity towards both the endothelial cell line bEnd.3 and murine glioma LCPN was found to be negligible. In vivo, the LCPN murine glioma animal model was used to evaluate BBB crossing, pharmacokinetics, and therapeutic effect. The system was able to reach the subjects’ brains after intravenous administration, and it targeted the tumour and extended morbidity-free survival in comparison to the untreated mice (16 days treated vs. 6 days untreated); it did not cause any obvious histological damage to major organs (kidney, spleen, liver, heart, or lung). A comparative study with a TMZ-treated control is, nonetheless, still necessary to evaluate performance against standard-of-care chemotherapy.

Todo et al. [106] conducted a single-arm phase II trial to test the effectiveness of intratumoural oncolytic herpes virus G47∆ for residual or recurrent GBM treatment. For nineteen patients with recurrent or residual GBM after treatment with surgery, TMZ and RT were selected and were administered G47∆ during stereotactic surgery. The maximum number of administrations was six and was limited by tumour progression. Median OS was 20.2 months and PFS was 4.7 months, which compare favourably with other treatments for recurrent GBM. All patients suffered from adverse effects related to immune responses, with more severe cases being managed with corticosteroids. These adverse effects were considered safe, and combined with the performance in this trial, G47∆ was approved in Japan on 11 June 2021, thus becoming the first oncolytic drug in Japan. This study was limited by a small sample size, and accompaniment of patients who received an apparent cure is still ongoing. 

Overall, although immunotherapy promises to deliver a very custom and targeted therapy to GBM, it has not yet delivered in late phase trials. There is still a lot of work to be done to overcome the immunosuppression hurdles that GBM presents and in developing preclinical models that more closely resemble the patients’ realities. Studies such as that by Todo et al., seem to be a potential step forward in creating a standard-of-care immunotherapy, but larger studies are necessary.

### 2.5. Tumour Treating Fields

**Figure 2:**
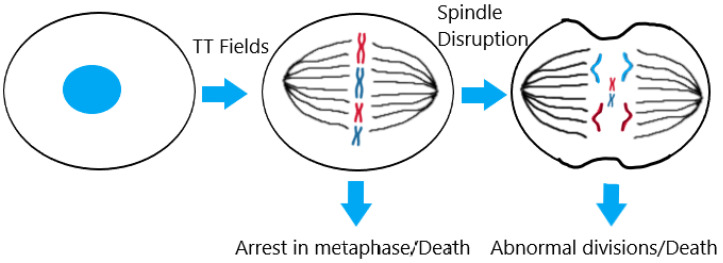
A simplified diagram of the effect of tumour treating fields (TTF) on proliferative cells.

TTF were approved by the FDA for treatment of recurrent GBM in 2011 (and for newly diagnosed GBM alongside TMZ in 2015) using low intensity (1–3 V/cm) and intermediate frequency (100–500 kHz) alternating electrical fields [9,70]. The new ASCO-NSO guideline recommends TTF therapy for newly diagnosed supratentorial glioblastoma after completing chemoradiation therapy [14]. The goal is to disrupt the cell cycle and halt cancer cell proliferation while not disturbing the normal functioning of quiescent cells [9,70]. These alternating electrical fields have since become standard of care in the United States, combined with surgery, radiotherapy, and chemotherapy [13]. This therapy results in increased OS without reducing quality of life, and adverse side effects are limited to skin reactions [11,67,70,107,108]. In practice, this treatment involves wearing on the shaved scalp a portable, patient-operated device with four transducer arrays for 18 h every day [77]. Normal proliferation of cancer cells gradually recovers after exposure to the TTF stops, hence the 18 h daily treatment duration [109]. The mechanism of action of TTF is based on the fact that the molecules and structures involved in cell division are highly polar and, as such, susceptible to applied electrical fields [110]. This means that, during metaphase, the formation of a functioning mitotic spindle is disturbed; during telophase, normal cytokinesis is disrupted; and during anaphase and telophase, the cellular shape combined with the field leads polar molecules to migrate towards the narrow furrow, thus disrupting the cell membrane [13,67,108,109,110]. A schematic of this mechanism is illustrated in Figure 2.

Besides disrupting cell division, it has been found that TTF can also induce apoptosis in cancer cells by either disrupting DNA repair mechanisms or triggering autophagic/immunogenic responses against these replicating cells [111]. 

This system is not without limitations; its cost is about $180,000 annually per patient [11,13,112]. Furthermore, most patients do not see their OS increase beyond 2 years [11]. Moreover, the efficacy of treatment is directly tied to patient compliance since treatment must be sustained and patient-operated [113]. To maximize the effectiveness and increase understanding of this treatment, there have been ongoing efforts to characterize TTF [114] with the aim of personalized care [115] and to augment the exposure of tumour areas to therapeutic doses of TTF via personalized array distributions [116]. There are also efforts to further optimizing the equipment’s running temperature for increased quality of life for the patient [117]. Nonetheless, according to EANO guidelines, the usage of this treatment modality remains controversial in its cost-effectiveness and feasibility as a standard of care, and, as such, it has seen reduced adoption [15]. Furthermore, these guidelines argue that the clinical benefit of TTF has not been established yet, which contradicts the ASCO-NSO guidelines and does not recommend this line of treatment in post-chemoradiation-therapy patient maintenance [15]. 

### 2.6. Photothermal and Photodynamic Therapy

Photothermal therapy (PTT) and PDT are treatments that rely on light sources, typically lasers, to achieve their effects [118,119]. In PTT, an NIR laser irradiates a tumour topically or through an optic fibre probe, which excites a photoabsorbing agent (PTA). This agent converts the irradiated light into heat, creating a localized hyperthermal effect that triggers cell death [118,120]. Depending on the target temperature, cell death might be triggered by thermal ablation (45 °C), or cellular damage and tumour permeability (42–43 °C) via protein denaturation and disruption of cellular membranes [121,122]. The materials for PTAs vary from metallic nanostructures of gold and copper to carbon and graphene nanostructures, polymer nanoparticles, and so on; they are sensitive to different laser wavelengths [123,124,125]. PTT can be associated with unwanted damage to surrounding healthy tissues, but delimitation of the light beam can be achieved via optical systems or blocking with insulating materials, such as aerogels [126]. 

A concrete example of PTT usage is magnetic resonance-guided laser-induced thermal therapy (MRgLITT or LITT), with systems like Visualase or NeuroBlate [112]. In these minimally invasive systems, MRI is used to localise the tumour, and then an NIR laser is transmitted via optic fibres to the target tissue, thus heating it to a target temperature of 43 °C [112,127]. MR thermography allows for real-time evaluation of temperature changes and ablation [127,128]. Treatment can still incur thermal damage to critical and eloquent brain tissue even with MRI guidance [128]; however, with proper training, it has potential to provide comparable survival to open surgery in recurring disease and provide a tumour removal option to patients’ ineligible for surgery [127,129,130,131].

Recently, results from a single-arm clinical trial (NCT03020017) [132] utilizing spherical nucleic acid (SNA) gold nanoparticles targeting the oncogene *BCL2L12* (B cell lymphoma 2-like 12) in recurrent GBM were released. Eight adult patients with recurrent GBM were treated with intravenous administration of these particles (named NU-0129), followed by tumour resection. NU-0129 was found to reach the tumour, incorporate into glioma cells, and induce reduction in *BCL2L12* protein expression. Nicole et al. [133] studied Prussian blue nanoparticles (PBNPs) conjugated with anti-fibroblast growth factor inducible 14 (anti-Fn14) for targeted photothermal therapy for GBM. Since Fn14 is a receptor abundantly expressed on many glioblastomas but near absent on healthy CNS tissue, the authors aimed to avoid off-target effects of PTT by targeting this receptor with their system. The conjugated system maintained its stability for at least 21 days, and in vitro PTT of U87MG and U251 resulted in reduction of cell viability from 97.85% to 18.45% and 95.00% to 11.19%, respectively. Furthermore, anti-Fn14-PBNPs exhibited higher cell attachment when the particles were incubated with U87MG cells, whereas simple PBNPs did not. In vivo tests were not conducted and are necessary to confirm targeted therapy in a live model.

In PDT light is used to activate a photosensitizer (PS) molecule responsible for the creation of ROS, which react with the macromolecules that constitute cell and organelle membranes, thus damaging these structures and inducing cell death [119,134]. It is oxygen dependent, meaning that adequate tissue oxygenation is necessary to achieve therapeutic effects [135]. As in PTT, NIR light has deep penetration and higher safety compared to shorter wavelengths that can be used, and PS drugs are also varied and include organic and inorganic nanostructures. These can be sensitive to specific wavelengths, include molecules that increase tissue oxygenation (e.g., catalase and haemoglobin), and can be functionalized with ligands higher for tumour tissue specificity [121]. Recently, Silva and colleagues [136] developed X-ray-absorbing scintillating europium-doped gadolinium oxide nanoparticles. Their goal was to create particles capable of converting X-ray radiation to ultraviolet-visible (UV-Vis) light for more efficient activation of PS. Both gadolinium oxide and hybrid silica and gadolinium oxide nanoparticles agglomerated in aqueous solution; however, they exhibited low to no toxicity in vitro, and were capable of converting X-rays to light in the UV-Vis range. Schipmann et al. [137] tested the use of 5-ALA, a fluorescent agent used for fluorescence-guided resection, as a PS precursor for PDT for recurrent high-grade glioma treatment. After removal of non-eloquent fluorescent tissue, diffusors were introduced in the cavity for laser irradiation of the remaining tissue (635 nm, 200 mW/cm diffusor). The combined regimen did not result in adverse events or new neurological deficits, and it delivered favourable PFS. However, a small sample size, retrospective design of the study, and difficulty in evaluating the effect of PDT in post-op MRI were limitations that warrant further studies to confirm their method’s viability.

### 2.7. Magnetic Thermotherapy

Like PTT, magnetic thermotherapy aims to induce localised cell death via an increase in temperature, either in hyperthermia or ablation ranges [138]. This is achieved via injection of superparamagnetic iron oxide nanoparticles (SPIONs) into the target tissue and exposure to an alternating magnetic field (AMF) [139,140]. Besides the more obvious impact in tissue disruption or ablation, enhanced infiltration of natural killer (NK) cells, T cells, dendritic cells, and macrophages into necrotic areas has been observed in preclinical glioma models [141]. Hyperthermia was also seen to induce an immune response chaperoned by heat shock protein (HSP)-peptide complexes released by dying glioma cells [142] These proteins are overexpressed in gliomas and are possible targets of inhibitors for hyperthermia sensitization; moreover, their release by dying cells stimulates T cell responses [141,142,143]. As in PTT, particle characteristics such as core size, composition, shape, and surface shell can be tailored to the application, and, as such, there have been systems designed for synergistic strategies with radio-, chemo-, and immunotherapy [138,140,141,144]. 

Recent studies include the work by Minaei and colleagues [145], where theragnostic folic-acid-conjugated TMZ-loaded SPION@poly(ethylene glycol)–poly(butylene adipate)–poly(ethylene glycol) (PEG-PBA-PEG) nanoparticles (TMZ-MNP-FA NPs) were synthesized to use as MRI contrast agents and enhance hyperthermia and radiotherapy. Particles exhibited a spherical shape, low polydispersity (PdI = 0.26), and a maximum size under 50 nm. TMZ release was pH dependant, increasing release at lower pH typical of tumour environments, and the system was capable of increasing temperature of solutions in a concentration-dependant fashion. Irradiation with AMF also increased the release rate of TMZ (6.4% at 37 °C to 55.2% at 37 °C after 10 min). Reduced cell viability with the nanosystem was also dose and time dependant when compared to TMZ alone, which was only dose dependant. Furthermore, the effects of TMZ-MNP-FA NPs + RT + AMF were triple those of the NPs alone, and this triple modality (thermo-chemo-radiotherapy) achieved a 2.59-fold increase in cell death in C6 cells. 

Basina et al. [146] synthesised Fe_3_O_4_ nanoparticles “decorated” with LAPONITE^®^ nanodisks, a synthetic clay with diamagnetic properties. The hybrids exhibited differing sizes, while the zeta potential varied in a Fe_3_O_4_-content-dependant fashion, decreasing as iron content increased. Particles with 50 wt% iron content were found to be the most efficient at inducing hyperthermia, with the highest specific absorption rate (SAR) at least 1.5 times higher than hybrids with other iron concentrations (540 W/g @ 150 kHz). The 50 wt% hybrids exhibited a mean size of 202.6 nm and a zeta potential of −31.6 mV, achieving good colloidal stability in water. In vitro incubation with human glioblastoma (U87-*EGFRvIII*) and human fibroblast (HFF1) showed no difference in cell survival or proliferation compared to the negative control. Exposure of the hybrid to a 150 kHz AMF after 24 h incubation resulted in a large drop in cell viability compared to the control in all cell types, including therapy-resistant glioblastoma variants U87-*EGFRvIII* and U87-*EGFR wildtype*. In in vivo imaging studies, the hybrids were observed to cause a signal drop on MRI *T_2_*-weighted imaging and persisted in the rodent brain for 7 days after convection-enhanced delivery (CED). Further studies to evaluate therapeutic performance in vivo are necessary.

## 3. Clinical Studies and the Most Recent Developments

In the previous sections, references to clinical studies and other recent works were provided to further contextualize the successes and challenges in each “standard-of-care” approach to GBM. This section aims to display the most representative (not all) clinical trials (Table 1) and additional developments (Table 2) of GBM treatment. 

## 4. Conclusions

The current standard of care for GBM combines various treatments: surgery, radio- and chemotherapy, and TTF. However, these treatments are not only incompletely effective but also boast low median survival, urging researchers and physicians to find new alternatives. The standard-of-care treatment has been upgraded from concomitant TMZ and radiotherapy with TTF since their approval by the FDA in 2015, but the 2-year OS remains the biggest hurdle to overcome. 

Advancements in preoperative and intraoperative medical imaging have improved the extent of tumoral tissue removal by allowing better surgical planning and increasing visual information and clarity of tumour tissue extension during surgery. Improvements in these techniques and the application of multiple imaging techniques have been paramount in improving surgical success and reducing damage to eloquent brain tissue. Unfortunately, resection alone does not complete tumour cell removal, resulting in the eventual manifestation of recurrent malignant masses. There has been work in refining the surgical procedure itself, gauging the importance of the extent of resection, and finding ways to increase both total resection and conservation of eloquent areas.

Treatment after maximal resection has also seen thorough research, with studies in both chemo- and immunotherapy seeking improvements in patient responses and OS. So far, no attempts have achieved significant success, though new studies and strategies are in continuous development. The preclinical results of immunotherapy have generally not been translated to success in human trials, and at least some of those struggles are attributed to GBM’s ability to adapt and avoid immune responses and the difficulty of creating models that closely and holistically represent the complexity of the immune system. That being said, recent successes might soon change this reality. As for chemotherapy, nanotechnological approaches have been developed to reduce side effects of antitumoral drugs and increase their BBB permeability, which is one of the main obstacles to drug availability at GBM sites. Alternatives, such as PTT and PDT, promise a targeted therapy against cancer cells while avoiding the side effects of radio- and chemotherapy. More work is necessary to validate their usefulness against GBM, as large-scale human trials are lacking. Herein, both preclinical and clinical trial successes have not translated to an increase in OS yet; it will take further innovation in the field to produce strategies capable of becoming the standard of care.

## Figures and Tables

**Table 1:** Clinical trials on GBM treatment.

Clinical Trials		
Materials/Technique	Main Outcomes	Ref.
Proton therapy (PT) X-ray radiotherapy (XRT)	Males and individuals treated with PT had lower incidence of radiation-induced grade 3+ lymphopenia (G3 + L) (15 and 14%, respectively) versus X-ray radiotherapy (XRT) (39%). Individuals treated with PT had lower a whole-brain mean dose (20.1 ± 5.7 Gy) compared to XRT (27.0 ± 6.1 Gy).	[93]
Intensity-modulated radiotherapy (IMRT) Photon therapy (PT)	No statistical difference in OS (median 21.2 months in IMRT vs. 24.5 months in PT), PFS (8.9 months in IMRT vs. 6.6 months in PT), or rates of deterioration between the two arms at 6 months. Reduction in mean number of toxicities (1.15 IMRT vs. 0.35 PT) and patient-reported fatigue (58% IMRT vs. 24% PT).	[94]
Ofranergene obadenovec (VB-111) + Bevacizumab Bevacizumab	No statistically significant improvement in OS was observed in recurrent GBM in VB-111 + bevacizumab versus bevacizumab monotherapy. Higher rate adverse events in the combination arm (67% vs. 40%).	[102]
Nivolumab Bevacizumab	Median OS was comparable between nivolumab (9.8 months) and bevacizumab (10 months). Safety profile of nivolumab in patients with glioblastoma was consistent with that in other tumour types.	[103]
Oncolytic herpes virus G47∆	Median OS was 20.2 months and PFS was 4.7 months. All patients suffered from safe adverse effects related to immune responses.	[106]
Spherical nucleic acid (SNA) gold nanoparticles (NU-0129)	NU-0129 was found to reach the tumour, incorporate into glioma cells, and induce reduction in *BCL2L12* protein expression. Four out of eight patients experienced adverse effects related to NU-0129, but there were no serious adverse effects.	[132]
Ad-RTS-hIL-12 (Ad) Veledimex (V) Nivolumab (nivo)	Treatment induced pseudoprogression with reduction of tumour size. Ad + V monotherapy increased peripheral T cells, unlike nivo monotherapy. Adverse effects consistent with their previous studies of Ad + V, being manageable and reversible. No OS data published yet.	[147]
Pembrolizumab Bevacizumab	Monotherapy and combination therapy were well tolerated. Pembrolizumab was ineffective for GBM treatment both as monotherapy and when combined with bevacizumab. OS below 11 months for both cohorts.	[148]
In vitro-expanded autologous CMV-specific T cells	No evidence of toxicity related to adoptive cell therapy (ACT). 10 of 25 patients alive at follow-up, 5 of which were progression-free. Median OS 21 months and PFS 10 months, increased to median 23 OS and 14 PFS if treated before recurrence.	[149]
Ipilimumab (IPI) Nivolumab (nivo)	Infrequent and mild immune-related adverse effects. Median PFS of 11.7 weeks and OS 38 weeks (<10 months). Treatment after maximal safe resection of recurrent GBM was safe, and increased OS.	[150]

Abbreviations: ACT, adoptive cell therapy; Ad + V, Ad-RTS-hIL-12 + Veledimex; CMV, cytomegalovirus; G3 + L, radiation-induced grade 3+ lymphophenia; GBM, glioblastoma multiforme; GSCs, glioblastoma stem cells; IMRT, intensity-modulated radiotherapy; IPI, Ipilimumab; OS, overall survival; PDT, photodynamic therapy; PFS, progression-free survival; PT, photon therapy; SNA, spherical nucleic acid; VB-111, ofranergene obadenovec; XRT, X-ray radiotherapy.

**Table 2:** Additional developments on GBM treatment.

Additional Developments		
Materials	Main Outcomes	Ref.
Core: poly(ethylene glycol) diacrylate (PEGDA) + Lanthanide-Silica Upconversion nanoparticles (UCNPs) Surface: fluorinated ethylene propylene (FEP)	Implants exhibited upconversion behaviour even when implanted in synthetic tissue. Increased apoptosis marker production (ROS and caspase 3/7) during PDT. Transdermal (wireless) capabilities confirmed on mouse model and on macaque brain.	[151]
PDT induced opening of the blood–brain barrier permeability (Laser: 635 nm 10–40 J/cm^2^, 40–100 mV)	PDT can impair brain fluid drainage/promote leakage and oedema. The meningeal lymphatic system is recruited when the BBB is opened. Managing this can allow for PDT procedures with reduced oedema. Optical coherence tomography (OCT) can be used to monitor drainage.	[152]
Pluronic (F-127) + curcumin (Cur) and/or CbV Theranostic photonic nanoparticles (TPNs)	Efficient BBB crossing of cur and CbV, attributed to the delivery by Pluronic-based TPNs. Efficient brain delivery and preferential targeting of GBM in a mouse model. 9-fold reduction of GBM proliferation and 1800-fold reduction of dose compared to free Cur. GBM regression.	[153]
Talazoparib, a PARP inhibitor (PARPi) TMZ	PARPi + TMZ + 4 Gy caused 36,6% cell cycle arrest (G2/M transition) at 72 h. This combination also obtained greater reduction of cell proliferation, and eliminated up to 97% of GSCs. Talazoparib achieved comparable or higher radiosensitization compared to other PARPis.	[154]
Dichloroacetate (DCA)	DCA increased mitochondrial ROS, induced apoptosis in GBM and GSCs, and depolarized mitochondria both in vitro and in vivo. Maximum dose (reversible neuropathy) induced no hematological, hepatic, renal, or cardiac toxicity.	[155]
DCA	DCA injection increased amine and amide concentration-independent detection (AACID) activity, corresponding to reduced intracellular pH (pHi). A single dose of DCA (200 mg/kg) was enough of a pharmacological challenge to reduce pHi.	[156]
NaDCA MgDCA	In vivo treatment with both NaDCA and MgDCA reduced tumour invasion. This reduction depended both on the concentration, the cation, and the glioma cell line (U87MG or PBT24). Xenograft growth and tumour vascularization were also dependent on concentration, cation, and cell line. NaDCA monotherapy was successful against the U87MG cell line, while MgDCA outperformed NaDCA against PBT24. These different performances may point towards relevant differences in the cell lines’ biology.	[157]
Drug combination: Metformin DCA Memantine	All treatment combinations (memantine, DCA, metformin + DCA) induced dose-dependent cytotoxicity in both tested cell lines (T98G and U87MG). The Alzheimer’s disease drug memantine might be used as GBM therapy at lower doses (almost 10 times lower) than metformin. Clinical trials are necessary to validate its viability.	[158]

Abbreviations: AACID, amine and amide concentration-independent detection; BBB, blood–brain barrier; CbV, carbazole arylvinyl [159]; Cur, curcumin; DCA, dichloroacetate; FEP, fluorinated ethylene propylene; GBM, glioblastoma multiforme; GSCs, glioblastoma stem cells; MgDCA, magnesium dichloroacetate; NaDCA, sodium dichloroacetate; nivo, Nivolumab; PARPi, Poly (ADP-ribose) polymerase inhibitor; PEGDA, poly(ethylene glycol) diacrylate; PDT, photodynamic therapy; PFS, progression-free survival; pHi, intracellular pH; ROS, reactive oxygen species; SNA, spherical nucleic acid; TMZ, Temozolamide; TPNs, Theranostic photonic nanoparticles; UCNPs, upconversion nanoparticles.

## Data Availability

No new data were created or analyzed in this study. Data sharing is not applicable to this article.

## Abbreviations

5-ALA	5-aminolevulinic acid
AACID	Amine and amide concentration independent detection
ACT	Adoptive cell therapy
Ad+V	Adaoviral-rheoswitch therapeutic system-human interleukin 12 + Veledimex
ALC	Absolute lymphocyte count
AMF	Alternating magnetic field
ASCO-SNO	American society of clinical oncology and the society for neuro-oncology
BBB	Blood-brain barrier
BOLD fMRI	Blood oxygenation level dependent functional magnetic resonance imaging
CbV	Carbazol arylvinyl
CED	Convection enhanced delivery
CHC	Alpha-cyano-4-hydroxycinnamic acid
CMV	Cytomegalovirus
CNS	Central nervous system
CT	Computed tomography
CTLA-4	Cytotoxic T-lymphocyte-associated protein 4
CTX	Cetuximab
Cur	Curcumin
CW	Carmustine wafer
DCA	Dichloroacetate
DESI-MS	Desorption electrospray ionisation mass spectrometry
DNA	Deoxyribonucleic acid
EANO	European association of neuro-oncology
FA	Folic acid
FDA	Food and drug administration
FEP	Fluorinated ethylene propylene
FET	^18^Fluoro-O-(2) fluoroethyl-l-tyrosine
Fn-14	Fibroblast growth factor inducible 14
G3+L	Radiation induced grade 3+ lymphopenia
GBM	Glioblastoma multiforme
GSCs	Glioblastoma stem cells
Gy	Gray
HF RT	Hypofractionated radiotherapy
HSP	Heat shock protein
ICG	Indocyanine green
IDH	Isocitrate dehydrogenase
IDO	Indoleamine-pyrrole 2,3-dioxygenase
IL	Interleukine
iMRI	Intraoperative magnetic resonance imaging
IMRT	Intensity-modulated radiotherapy
IPI	Ipilimumab
KPS	Karnofsky performance score
MgDCA	Magnesium dichloroacetate
MGMT	O6-methylguanine deoxyribonucleic acid methyl-transferase
MNP	Magnetic nanoparticle
MPC	2-methacryloyloxyethyl phosphorylcholine
MRgLITT	Magnetic resonance-guided laser-induced thermal therapy
MRI	Magnetic resonance imaging
MRS	Magnetic resonance spectroscopy
NaDCA	Sodium dichloroacetate
NIR	Near-infrared
nivo	Nivolumab
NK	Natural killer
NPG	Nanoparticle gel
OCT	Optical coherence tomography
OS	Overall survival
PARPi	Poly-(adenosine diphosphate ribose) polymerase inhibitor
PBA	Poly(butylene adipate)
PBNP	Prussian-blue nanoparticle
PBRT	Proton beam radiation therapy
PdI	Polydispersity index
PDL1-siRNA	Programmed death-ligand 1-small interfering ribonucleic acid
PDT	Photodynamic therapy
PEGDA	Poly(ethylene-glycol) diacrylate
PEGMA	Poly(ethylene-glycol) methacrylate
PET	Positron emission tomography
PFS	Progression-free survival
PLGA	Poly(lactic-co-glycolic acid)
pHi	Intracellular pH
PROs	Patient reported outcomes
PsP	Pseudoprogression
PS	Photosensitizer
PT	Photon therapy
PTA	Photoabsorbing agent
PTX	Paclitaxel
RANO	Response assessment in neuro-oncology
ROS	Reactive oxygen species
SAR	Specific absorption rate
SNA	Spherical nucleic acid
SPION	Superparamagnetic iron oxide nanoparticles
SRS	Stereotactic rradiosurgery
TMZ	Temozolomide
TP	Tumour progression
TPN	Theranostic photonic nanoparticles
TSPO	Translocator protein
TTF	Tumour treating fields
UCNPs	Upconversion nanoparticles
USPIONs	Ultra-small iron oxide nanoparticles
UV-Vis	Ultraviolet-visible
VB-111	Ofranergene obadenovec
VEGF	Vascular endothelial growth factor
WBMD	Whole-brain mean dose
WHO	World health organization
XRT	X-ray radiotherapy
